# Legal consciousness revisited: a dynamic framework for rights awareness in a relational and mediated world

**DOI:** 10.3389/fsoc.2026.1697330

**Published:** 2026-05-20

**Authors:** Alfonso Renato Vargas Murillo, Hugo Adan Falconi Tupiño

**Affiliations:** Facultad de Derecho y Ciencias Políticas, Universidad Privada del Norte, Lima, Peru

**Keywords:** digitalization, legal consciousness, relational theory, rights consciousness, social mobilization, sociology of law, theoretical framework

## Abstract

While foundational, classic legal consciousness scholarship is insufficient to capture the complexity of contemporary citizen-law relations. This article distinguishes legal consciousness from rights consciousness, treating the latter as the claim-oriented subset of legality-making concerned with entitlement, remedy, and institutional redress, and proposes a dynamic, multi-layered framework for analyzing their interaction. Synthesizing classic and recent socio-legal work, the framework is organized into three interconnected layers: (1) a foundational layer, which recasts legality as relational, plural, and cognitively mediated; (2) a mobilization and resistance layer, which theorizes rights mobilization, strategic withdrawal, and legal insufficiency in relation to adjacent concepts such as legal alienation, legal estrangement, and legal cynicism; and (3) a contemporary contextual layer, which defines digital legal consciousness through online dispute navigation, algorithmic curation and affect, platform governance, and digitally networked rights-claiming, thereby showing how digital legal consciousness reshapes and partially decouples legal sense-making from national legal cultures, while specifying the scope conditions under which geopolitical comparison and cultural intimacy matter most—especially in semi-peripheral and post-transitional settings. To facilitate empirical use, the article also maps the framework's three cross-layer mechanisms—mediation, feedback, and re-framing—to concise scope conditions, observable indicators, and exemplary literatures. The result is an analytically sharper tool for explaining when legality is experienced as claimable, insufficient, estranging, or strategically withdrawable in an increasingly interconnected and mediated world.

## 1 Introduction

The relationship between citizens and the law is being reworked under conditions of digital transformation, political polarization, and declining institutional trust. Legal consciousness is the scholarly concept that captures this interaction, describing the patterns of thought and action through which people understand the world from a legal perspective (Ewick and Silbey, 2005). The classic paradigm established by Ewick and Silbey (1998)—with its tripartite narrative structure of “before the law,” “with the law,” and “against the law”—has been indispensable for explaining the persistence of legal hegemony (Halliday and Morgan, 2013). Yet as the field has matured, the model's explanatory limits have become clearer, especially when scholars seek to explain how legal consciousness shifts across institutions, movements, digital environments, and historical conjunctures (Cowan, 2004; Chua and Engel, 2019).

New social and technological forces are drastically reshaping this relationship. Digitalization is altering the very structure of legal thought and socialization, giving rise to an emergent digital legal consciousness—that is, patterns of legal sense-making and rights navigation shaped primarily by digital platforms and online justice interfaces—that reshapes and partially decouples those practices from national legal cultures (Creutzfeldt, 2023). This consciousness is not formed in a vacuum; it is actively shaped by the affective tone of media coverage following key judicial decisions (Bailey et al., 2025) and is profoundly mediated by political trust, which itself is influenced by algorithmically-driven information exposure (Peng et al., 2025). Globalization and geopolitical positioning introduce a reflexive, comparative dimension to legal consciousness, particularly in nations on the periphery of hegemonic centers. Here, the often-painful gap between international legal ideals and the perceived dysfunctionality of local practice is managed through what Fossum (2025) terms cultural intimacy: a rueful, ironic self-recognition of systemic failings as somehow essential to the national identity.

Furthermore, mass political mobilization and profound institutional dissatisfaction are reconfiguring citizens' engagement with the law. Participation in protests can generate a tangible empowerment effect—a mobilization-induced increase in perceived efficacy and willingness to claim formal rights—(Hilbink and Salas Ramos, 2024). Deep antipathy toward the state does not necessarily produce legal alienation but can instead manifest as a critique of legal insufficiency—a frame in which institutional dysfunction is understood as the under-application or under-enforcement of law, prompting demands for a more robust realization of legality—even toward institutions citizens simultaneously distrust (Feddersen et al., 2024). Finally, for marginalized populations such as sexual and gender minority (SGM) unaccompanied minors, legal consciousness is not a static position but a biographical and evolving process, forged through traumatic encounters with the state that teach them their rights are contingent on their legal status (Tenorio, 2025).

This article proposes a multi-layered conceptual framework that moves beyond a descriptive restatement of the literature to theorize the dynamic, relational, and often paradoxical nature of rights consciousness. Synthesizing insights from studies on migration, post-socialist transitions, political mobilization, digitalization, and institutional dissatisfaction, the paper constructs a framework organized into three interconnected layers: (1) the Foundational Layer, which reconstructs sociological narratives as relational and cognitively mediated; (2) the Mobilization and Resistance Layer, which explains how empowerment, strategic withdrawal, and legal insufficiency emerge; and (3) the Contemporary Contextual Layer, which specifies how digital mediation, geopolitical comparison, and biographical temporality reshape legal sense-making. The aim is not merely to redescribe how people experience law, but to explain when they claim, contest, defer, or reframe their rights.

Two definitional clarifications are necessary at the outset. First, this article does not treat legal consciousness and rights consciousness as interchangeable terms. Legal consciousness refers to the broader repertoire through which people perceive legality, authority, obligation, and institutional order in everyday life (Ewick and Silbey, 1998; Chua and Engel, 2019). Rights consciousness is used here more narrowly to designate the claim-oriented subset of that repertoire: the ways actors recognize injuries as rights violations, imagine remedies, and decide whether to mobilize, defer, or strategically suspend claims (Scheingold, 2004; McCann, 1994). The distinction matters because actors may remain deeply legality-oriented while not activating rights claims, or may translate grievances into rights talk only under particular organizational, relational, and political conditions.

The paper is positioned in three debates that are central to contemporary sociology of law. First, it intervenes in the debate over whether legal consciousness should be treated primarily as a narrative typology or as a relational process produced through encounters with institutions, adversaries, and imagined communities (Ewick and Silbey, 1998; Chua and Engel, 2019; Liu, 2026; Young and Chimowitz, 2022). Second, it speaks to the debate over whether dissatisfaction with law produces alienation from legality or instead a re-politicized, ambivalent investment in law through claims, critique, and demands for enforcement (Gallagher, 2006; Hertogh, 2018; Feddersen et al., 2024). Third, it addresses whether legal consciousness can still be analyzed mainly within national legal cultures or must now be understood through digital infrastructures, transnational comparison, and temporally layered biographies (Creutzfeldt, 2023; Fossum, 2025; Tenorio, 2025).

From these debates, the article derives five scope-conditioned propositions that are theoretically testable. Proposition 1: algorithmically mediated exposure is more likely to depress legality-oriented attitudes when it is affectively polarizing, weakly anchored in credible institutional pathways, or experienced through opaque moderation; it is more likely to strengthen claim-oriented legality when credible institutional mediation, usable digital justice pathways, or trust-enhancing intermediaries are present (Peng et al., 2025; Katsh and Rabinovich-Einy, 2017; Pasquale, 2015). Empirically, this proposition requires attention to endogeneity and selection, since actors do not encounter platforms, official channels, or moderation environments at random. Proposition 2: collective mobilization reshapes foundational narratives most strongly when protest participation is coupled with rights framing, organizational support, and practical knowledge of where to turn, thereby transforming grievance into efficacy and claim-making (McCann, 1994; Albiston, 2005; Hilbink and Salas Ramos, 2024). Proposition 3: under conditions of institutional disappointment, actors frame grievances as legal insufficiency rather than legal alienation, legal estrangement, or legal cynicism when they retain normative commitment to legality while perceiving selective under-enforcement, selective impunity, or procedurally unjust implementation (Bell, 2017; Sampson and Bartusch, 1998; Tyler, 2006; Feddersen et al., 2024). Proposition 4: in semi-peripheral or post-transitional settings marked by sustained comparison to external legal models, geopolitical benchmarking and cultural intimacy intensify legality-oriented critique more than withdrawal when transnational norms remain aspirational and locally translatable rather than wholly rejected (Fossum, 2025; Teubner, 1998; Risse et al., 1999). Proposition 5: repeated encounters with coercive, welfare, or migration institutions shift legal consciousness over time, producing movement between claiming, strategic withdrawal, and renewed claiming as biography, status security, and organizational supports change (Hirschman, 1970; Engel and Munger, 2003; Tenorio, 2025).

**Figure 1:**
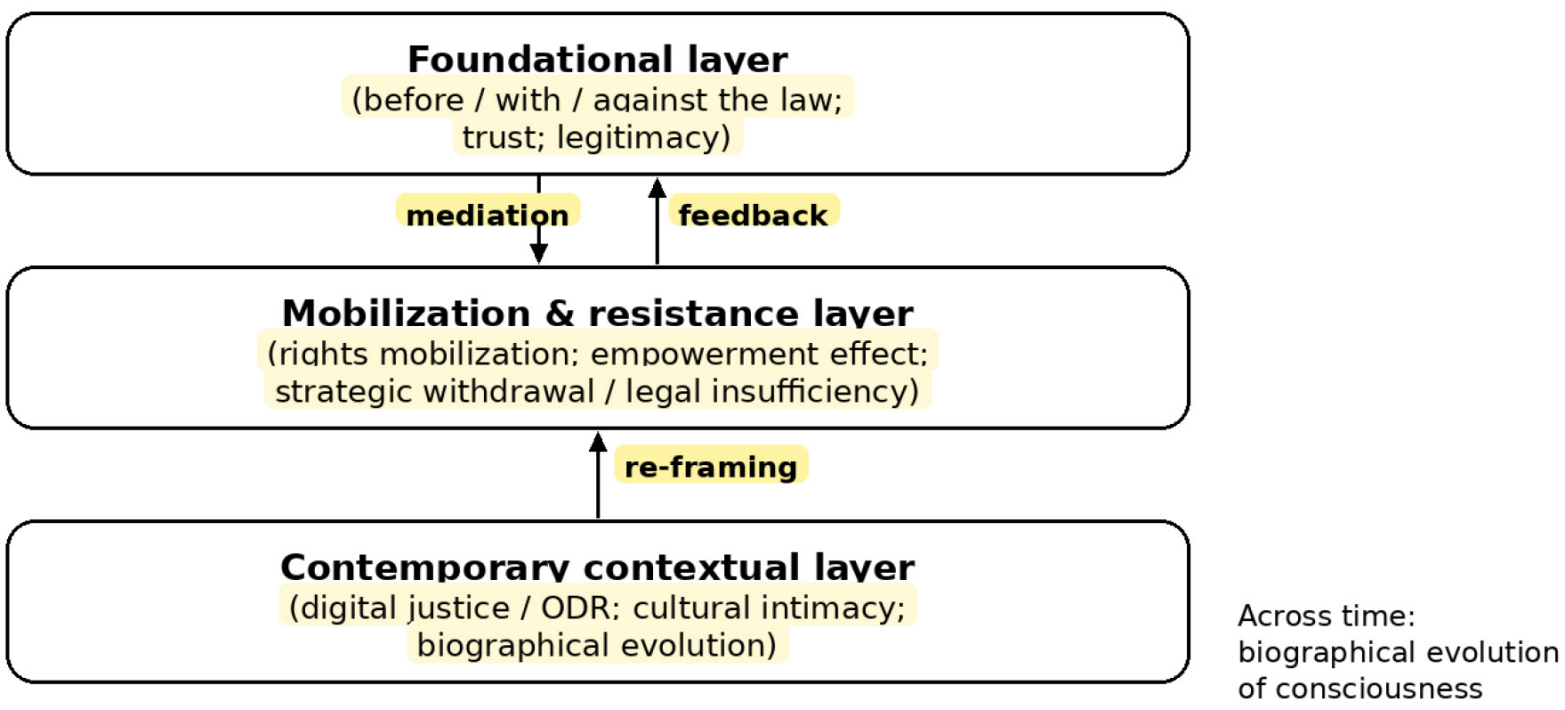
Dynamic, multi-layered framework for rights consciousness.

The contribution is therefore not a new typology but a mechanism-guided architecture that clarifies when and why similar foundational narratives yield divergent strategies of claiming, withdrawal, and critique. The article proceeds as follows. Section 2 reconstructs the foundational layer and specifies the mediation mechanism linking cognition, affect, relational narratives, and plural normative settings. Section 3 develops the action layer and shows how mobilization, organizational support, and institutional disappointment generate feedback into subsequent legal meaning-making. Section 4 identifies the contemporary conditions—digital infrastructures, geopolitical comparison, and biographical temporality—that re-frame both narratives and strategies. To facilitate empirical operationalization, Table 1 maps each cross-layer mechanism onto concise scope conditions, observable indicators, and exemplary literatures, while Figure 1 provides a schematic overview of the framework.

**Table 1:** This table translates the framework's three cross-layer mechanisms into a compact set of scope conditions, observable indicators, and exemplary literatures.

Mechanism	Scope conditions	Observable indicators/measures	Exemplary literatures
Mediation	Algorithmic exposure, credible institutional channels, usable ODR/e-justice entry points, platform moderation experience.	Primary source of legal information; exposure to official channels; moderation experiences; trust in courts/state; ODR or legal-tech use; perceived fairness/opacity; willingness to claim vs withdraw.	Katsh and Rabinovich-Einy, 2017; Sela, 2018; Sandefur, 2019; Peng et al., 2025; Papacharissi, 2014; Pasquale, 2015.
Feedback	Protest participation plus rights framing, organizational support, practical knowledge of where to turn, institutional uptake.	Participation in protest/organizations; efficacy; naming grievances as rights; repeat claims; changes in official practice.	McCann, 1994; Scheingold, 2004; Albiston, 2005; Hilbink and Salas Ramos, 2024; Halliday and Carruthers, 2007; Velasco Domínguez, 2024.
Re-framing	Semi-peripheral/post-transitional settings, sustained comparison to external legal models, repeated state encounters, status precarity.	Comparative references to foreign legality; narratives of national failure/cultural intimacy; exit/voice/strategic withdrawal; renewed claiming over time.	Watson, 1993; Teubner, 1998; Risse et al., 1999; Merry, 2005; Fossum, 2025; Hirschman, 1970; Tenorio, 2025.

The table is not intended as an exhaustive codebook; rather, it offers a bridge from conceptual architecture to mixed-method, longitudinal, and comparative socio-legal research designs.

Before developing the three layers, we clarify the conceptual approach and the logic used to select and integrate the empirical literature mobilized in this framework. This manuscript is a theory-building conceptual synthesis: it is not an original empirical study, and it does not claim the exhaustiveness of a systematic review.

The framework was developed through an iterative, purposive engagement with the legal consciousness literature aimed at maximizing theoretical leverage rather than exhaustive coverage. We prioritize (a) canonical contributions that define core socio-legal constructs and debates and (b) recent empirically grounded studies that illuminate one of the framework's three layers. Empirical studies are therefore used as mechanism-revealing exemplars to anchor conceptual claims and to derive the five propositions above; they are not used to support prevalence estimates or universal causal claims. This choice gives the article an explicit contribution pathway: conceptual reconstruction, mechanism specification, and proposition-building for subsequent empirical testing.

## 2 The foundational layer of legal consciousness: a relational construct

To build a robust conceptual framework, one must begin at its foundations. The foundational layer of legal consciousness rests upon the interplay between a society's legal culture, the sociological narratives through which legality is expressed, the cognitive structures that process it, and the philosophical debates that legitimize it. A theoretical reconstruction, however, must recognize that these elements are not merely internalized by isolated individuals but are co-constituted relationally (Chua and Engel, 2019).

The starting point is the distinction between legal culture—a collective phenomenon of attitudes and values shaped by history—and legal consciousness, its manifestation at the individual level (Szilágyi, 2023). For present purposes, however, a second distinction is equally important: legal consciousness names the broader everyday orientation to legality, whereas rights consciousness refers to the narrower readiness to interpret injury, need, or conflict through the idiom of entitlement, remedy, and institutional redress (Scheingold, 2004; McCann, 1994). This claim-centered subset does not float free of broader legality; it is activated, constrained, or deferred within what Moore (1973) called semi-autonomous social fields and within the overlapping normative orders emphasized by legal pluralism (Griffiths, 1986). The gap between legal culture and legal consciousness, such as the post-socialist limp where countries formally adopt Western legal structures but retain low institutional trust (Novak, 2023), demonstrates that consciousness is not a simple reflection of the “law on the books”. Within this culture, individual consciousness is expressed through the three classic narratives of Ewick and Silbey (1998): “before the law” (law as a majestic, objective entity), “with the law” (law as a strategic game), and “against the law” (law as an oppressive power).

However, the adoption of these narratives is a fundamentally relational act. As Liu (2026) argues, an individual's understanding of law is profoundly shaped by their encounters with perpetrators, the legal system, and the nation-state. A victim of a hate crime, for instance, does not experience the law in the abstract but in direct relation to their aggressor and their perception of whether the system will offer protection or inflict further burdens. This perspective reframes the classic schemas not as static psychological postures but as fluid, relational positions adopted and negotiated through social interaction (Young and Chimowitz, 2022). Such situated encounters also occur within institutions and organizations that teach people what kinds of claims are intelligible, risky, or worthwhile—from offensive public speech to workplace civil-rights mobilization (Nielsen, 2004; Albiston, 2005). This relational consciousness is also deeply connected to identity, as one's sense of self shapes and is shaped by these legal interactions (Engel and Munger, 2003).

This relational foundation is increasingly challenged and reconfigured by the digital sphere. Reviews of the field show that legal consciousness is not exhausted by formal legal knowledge; it also involves interpretive and cognitive capacities through which people evaluate legality in everyday life (Horák et al., 2021). Those capacities now operate in a non-neutral information environment. Research on Chinese university students by Peng et al. (2025) shows that information exposure in digital media can exert a direct negative effect on legal consciousness, attributable to algorithmic biases toward emotionally charged and ideologically selective content. Yet this negative effect is counterbalanced by indirect pathways: the same exposure, when channeled through credible sources, can bolster political trust, which in turn positively mediates legal consciousness. This finding is critical because it shows that the cognitive underpinnings of consciousness are no longer solely dependent on formal knowledge but on a media ecology that shapes affect and trust toward institutions.

Finally, the philosophical underpinnings of legal consciousness—the tension between a formal consciousness (belief in the law's binding validity) and a material consciousness (what individuals deem just) (Krešić, 2019), or the notion of a moral ideal as a criterion to critique positive law (Poole, 2022)—must also be understood relationally. An individual's perception of whether the Rule of Law is formal, substantive, or functional (Horák and Lacko, 2023) is filtered through their interactions with and trust in the institutions that embody it.

A critical interpretation of this layer reveals that legal consciousness emerges from the constant and dynamic interaction between macro-level forces (legal culture, ideologies) and micro-level processes (cognition, emotion), but this interaction is fundamentally relational, plural, and technologically mediated. A robust conceptual framework must acknowledge that the “Rule of Law” an individual perceives is molded by a historically contingent legal culture (Novak, 2023) and, increasingly, by a digital ecosystem that can simultaneously enhance and erode trust in that very system (Peng et al., 2025). It must also account for the fact that actors interpret legality across overlapping normative orders and vernacular translations, rather than through state law alone (Moore, 1973; Griffiths, 1986; Merry, 2005).

In other words, the first cross-layer mechanism is mediation: contemporary information environments modulate the cognitive and affective conditions under which classic narratives become plausible and actionable. The foundational layer therefore yields Proposition 1: digitally mediated exposure strengthens claims-oriented legal consciousness only where trust and credible institutional mediation convert information into efficacy; where exposure is affectively polarizing and distrusting, the same environment is more likely to reinforce cynicism, defensive pragmatism, or withdrawal. The next section moves from these mediated interpretive repertoires to the action layer, showing how strategies of claiming, withdrawal, and critique generate feedback that can, over time, recalibrate foundational meanings and expectations. Consciousness is neither purely determined by social structure nor an isolated product of individual psychology, but a co-constructed and evolving phenomenon.

## 3 The mobilization and resistance layer: from empowerment to legal insufficiency

Legal consciousness is not a passive state; it is manifested in action (Ewick and Silbey, 2005). Building on the foundational layer, this second layer specifies how mediated and relational narratives are converted into practical repertoires of mobilization, resistance, and claiming—and how action, in turn, feeds back into subsequent sense-making. Here, strategic withdrawal refers to a conditional suspension of voice under conditions of high risk or low institutional receptivity; unlike exit, it does not sever the relationship to law, and unlike generalized alienation, it preserves the possibility of renewed claiming when conditions change (Hirschman, 1970; Hertogh, 2018; Tenorio, 2025). In the classic rights-mobilization literature, rights are neither merely possessions nor guarantees; they are political resources and interpretive frames whose strategic value depends on organization, movement infrastructure, and institutional opportunity (Scheingold, 2004; McCann, 1994). A contemporary perspective must therefore move beyond a simple obedience/disobedience dichotomy to explore a spectrum of strategies that individuals and groups employ in a complex, often paradoxical, relationship with legal power.

The mobilization of law is marked by ambivalence, encapsulated in Merry's (1990) paradox of law, where recourse to the courts is simultaneously a source of empowerment and anxiety. This ambivalence is often worked through in specific institutional settings. Nielsen's (2004) analysis of offensive public speech and Albiston's (2005) account of workplace civil-rights mobilization show that organizations help script whether rights are perceived as usable, costly, or disruptive. This tension often leads not to inaction, but to forms of pragmatic resistance, such as the embedded resistance of migrant women who act covertly within the constraints of sociocultural pressures (Graca, 2021).

The decision to act is not always structurally determined. While studies in Norway show that social class significantly affects immigrants' legal consciousness and strategies (Leirvik et al., 2026), research in contexts of mass mobilization like Chile has observed that structural factors are not significant predictors of the willingness to claim rights (Hilbink and Salas Ramos, 2024). Instead, a practical factor such as the perception of knowing where to turn emerges as crucial.

Critically, individual resistance can scale into collective action, which in turn has a powerful feedback effect on individual consciousness. Research in Chile suggests that participation in protests can generate a significant empowerment effect (Hilbink and Salas Ramos, 2024). This finding is pivotal: mobilization is not merely the expression of a pre-existing legal consciousness but is a causal mechanism that can forge a new, more claims-oriented consciousness, even among traditionally disempowered groups.

This feedback can also reshape institutional legal consciousness. Feminist mobilization in Mexico has proven capable of transforming the legal consciousness of state actors themselves, such as prosecutors, influencing how they interpret and apply the law (Velasco Domínguez, 2024). This supports a recursive view of law, in which collective action, institutional uptake, and subsequent redesign interact in cycles rather than in one-directional diffusion (Halliday and Carruthers, 2007). However, dissent is not always progressive. The concept of dissident collectivism can be mobilized by groups to oppose laws protecting vulnerable minorities, often in cooperation with state actors (Gastrow, 2024).

In societies characterized by legal pluralism—where state law coexists with community, religious, organizational, and transnational normative orders (Moore, 1973; Griffiths, 1986; Jumarim et al., 2024)—the strategic navigation between forums is also a process of translation. Rights claims are vernacularized differently depending on what actors perceive as socially legible, emotionally credible, and institutionally reachable (Merry, 2005; Bens, 2024). When state law is perceived as ineffective, as with anti-corruption frameworks disconnected from local realities (Labik Amanquandor, 2024), a consciousness parallel to the law may emerge, where communities create their own governance systems (Gastrow, 2024).

This dynamic is complicated in contexts of deep institutional dissatisfaction. The research of Feddersen et al. (2024) in post-uprising Chile reveals a counterintuitive pattern. Despite pervasive antipathy toward the state, citizens do not exhibit legal alienation (Hertogh, 2018). Instead, they frame their dissatisfaction in terms of legal insufficiency. They critique institutions not for the existence of law, but for its failure to be applied rigorously, especially against corrupt elites.

The concept of legal insufficiency proposed here should therefore be distinguished from adjacent constructs. Unlike legal alienation, which denotes a more generalized sense that law is distant or not one's own (Hertogh, 2018), legal insufficiency remains legality-oriented: actors criticize institutions because law is selectively under-applied, not because legality has lost normative force. It also differs from legal estrangement, which emphasizes the collective experience of exclusion and state abandonment produced by coercive institutions (Bell, 2017), and from legal cynicism, which captures beliefs that law lacks binding or protective force in everyday practice (Sampson and Bartusch, 1998). Procedural justice research further suggests that distrust intensifies when institutions are experienced as unfair, disrespectful, or opaque, even when legality as an ideal remains valued (Tyler, 2006). Legal insufficiency thus names a narrower mechanism: critique directed at the weak realization of legality, rather than its wholesale rejection. Operationally, interview or survey instruments can distinguish this mechanism by pairing items on perceived under-enforcement or selective impunity with items on whether law should still bind everyone and whether legal remedies remain desirable.

This ambivalent stance—simultaneously rejecting institutions while demanding a stronger version of legality—shows that even in resistance, the law remains a central framework of meaning. This is a form of informed disenchantment where cynicism toward systemic failures coexists with a durable belief in the ideal of law (Gallagher, 2006).

Finally, resistance can manifest as strategic withdrawal, born from an evolving legal consciousness shaped by trauma. The work of Tenorio (2025) on SGM unaccompanied minors demonstrates how initial trust in the law as a protector is shattered by punitive experiences in state custody. This leads to a new understanding where rights are perceived as contingent on legal status. Consequently, these minors strategically withhold crucial information in their legal proceedings, a form of passive resistance aimed at survival by prioritizing the acquisition of papers over the immediate claiming of identity-based rights. In Hirschman's (1970) terms, this is not a simple abandonment of legality, but a strategic suspension of voice under conditions where immediate claiming is too costly or ineffective.

A critical analysis reveals that resistance is not a monolith. A robust framework must account for its varied forms: pragmatic vs. overt, individual vs. collective, progressive vs. regressive, and empowerment vs. strategic retreat. It must recognize that resistance is not always against the state, but can occur within its institutions (Haddeland and Franko, 2022) or even with its actors (Gastrow, 2024). The central paradox is that even in acts of resistance, actors often reinforce the centrality of law by engaging with its ideals or demanding its proper application. As Liu (2026) shows, for victims of hate crimes, the law is both a symbolic promise of protection and a source of burdens and harm. Legal consciousness, therefore, is not a simple binary of acceptance or rejection, but a continuous, conflictual, and often contradictory engagement with both the promise and the failure of law.

Crucially, these repertoires are not enacted in a vacuum: they are mediated by contemporary information environments and re-framed by comparative geopolitical imaginaries and biographical trajectories. The mobilization layer therefore supports two further propositions. Proposition 2 states that participation in collective mobilization can generate a more claims-oriented legal consciousness by increasing perceived efficacy and procedural know-how, not merely by expressing prior commitments (Hilbink and Salas Ramos, 2024). Proposition 3 states that institutional disappointment will be framed as legal insufficiency rather than legal alienation when actors continue to invest normatively in legality while perceiving selective under-enforcement or elite impunity (Gallagher, 2006; Hertogh, 2018; Feddersen et al., 2024).

## 4 The contemporary contextual layer: digitalization, geopolitics, and temporality

Legal consciousness is profoundly shaped by the social, political, and technological context in which it develops. This third layer does not come after the first two; it specifies the contemporaneous conditions that mediate foundational meaning-making and re-frame mobilization dynamics over time. The third layer of our framework focuses on contemporary forces that are reconfiguring the relationship between individuals, law, and rights, highlighting the impact of digitalization, the geopolitics of law, and the biographical evolution of consciousness.

### 4.1 Digital legal consciousness: an affective and mediated phenomenon

Digitalization represents not simply a new channel of communication but a reorganization of the infrastructures through which people search for remedies, interpret authority, and learn how law can be used. We define digital legal consciousness as patterns of legal perception, trust, dispute navigation, and rights-claiming that are formed through repeated interaction with digital platforms, online justice interfaces, legal-help tools, and platform-governed information ecologies. Analytically, the construct has four dimensions. First, it includes pathways into online dispute resolution, e-justice, and consumer-facing legal technologies that alter how users encounter procedure and fairness (Katsh and Rabinovich-Einy, 2017; Sela, 2018; Sandefur, 2019). Second, it includes algorithmic curation and affective amplification, through which exposure, salience, and political trust are patterned rather than neutrally distributed (Papacharissi, 2014; Gillespie, 2014; Pasquale, 2015). Third, it encompasses platform governance and content moderation, which shape the visibility, suppression, and perceived legitimacy of legal claims in public discourse (Ananny and Gillespie, 2016; Gillespie, 2018). Fourth, it includes digitally networked mobilization and connective action, where rights talk circulates through personalized and networked forms of protest (Bennett and Segerberg, 2012; Tufekci, 2017).

Within this expanded field, Creutzfeldt's (2023) work on post-pandemic disputing behavior suggests not a fully supranational break from national legal cultures, but a reconfiguration of how users experience justice when digital and legal capabilities become intertwined. Digital justice environments can widen access for some users while redistributing procedural burdens onto those with lower digital capacity or weaker institutional support (Katsh and Rabinovich-Einy, 2017; Sandefur, 2019). This helps specify why digital legal consciousness should be treated as a socio-legal formation rather than a mere technological trend.

However, the nature of this digital consciousness is neither neutral nor purely cognitive. As demonstrated by Peng et al. (2025), information exposure in algorithmic environments can have a direct negative effect on legal consciousness when emotionally polarizing or ideologically selective content dominates. Yet such exposure can also have an indirect positive effect when mediated by political trust and credible official channels. Bailey et al.'s (2025) analysis of judicial decisions and LGBTQ+ media likewise shows how affective tone and legal salience are jointly produced through mediated interpretation. These findings specify the mediation mechanism: digital environments shape legality not only by informing citizens, but by sorting attention, calibrating trust, and structuring whether claims appear plausible, risky, or worth pursuing.

### 4.2 Geopolitics, law, and the management of failure through “cultural intimacy”

In a legally interconnected world, legal consciousness, especially in countries outside Western hegemonic centers, acquires a reflexive and comparative dimension. This mechanism is not universal in the same way across all settings; analytically, it is strongest in semi-peripheral, post-transitional, or otherwise externally benchmarked contexts where actors routinely compare domestic legality to transnational models. That comparison can be understood in relation to legal transfer and diffusion literatures: imported norms may travel as transplants (Watson, 1993), operate as irritants that trigger unexpected local divergence (Teubner, 1998), or move through gradual domesticization and translation processes (Risse et al., 1999; Merry, 2005). The study by Fossum (2025) on intellectual property reform in Turkey is highly illustrative. Legal and industry actors constantly measure their domestic copyright system against an imagined, idealized “West”, revealing a heightened group-status awareness.

When local practice inevitably falls short of this international ideal, a dissonance emerges. Fossum argues that this gap is managed through cultural intimacy: a rueful self-recognition of systemic flaws that, while a source of external embarrassment, provides insiders with an assurance of common sociality (Herzfeld, 2005, p. 3). Complaints about bureaucracy or inefficiency become familiar narratives (“that's just how we are”) that explain the system's failures in terms of a shared national essence. This mechanism is critical: it allows actors to live with the contradiction between the aspiration for universal norms and the reality of their imperfect implementation, thereby sustaining the law's hegemony without requiring its flawless functioning. Under these scope conditions, re-framing is more likely to yield demands for stronger legality than outright withdrawal, because failure is narrated as a gap to be corrected rather than proof that law is irrelevant.

### 4.3 Humanitarianism, biopolitics, and bare life: the consequences at the border

Finally, legal consciousness is not a snapshot but a biography, evolving through direct and often formative experiences with the state.

The longitudinal research of Tenorio (2025) with SGM unaccompanied minors starkly illustrates this temporal evolution. Their consciousness transforms through distinct phases: an initial trust in U.S. law as a protector; a subsequent disillusionment after punitive encounters with state agents, leading to a new belief that their rights are contingent on legal status; and a final phase of empowerment and active rights-claiming post-legalization. This biographical perspective is essential, demonstrating how consciousness is shaped by a cumulative history of interactions with state power.

This process is also visible in how public policies, once institutionalized, can radically alter social perceptions, transforming a privilege into a right and a reparation, as seen with racial quotas in Brazil (Giraut, 2024). Furthermore, the concept of intimate citizenship highlights how the state's regulation of private matters like family and marriage becomes a crucial site for the formation and contestation of legal consciousness, particularly for migrants (de Hart and Besselsen, 2021).

A critical interpretation of this layer underscores that contemporary contexts accelerate and pattern the complexity of legal consciousness. Digitalization creates a translocal legal environment mediated by trust and affect (Creutzfeldt, 2023; Peng et al., 2025; Bailey et al., 2025). Geopolitical positioning forces actors to compare global standards with local realities, often relying on cultural intimacy to live with persistent gaps (Fossum, 2025). Biographical encounters with the state sediment these comparisons into durable expectations, fears, and strategies (Tenorio, 2025). This layer therefore specifies Proposition 4—that geopolitical comparison intensifies critique while cultural intimacy stabilizes compliance—and Proposition 5—that repeated encounters with coercive or protective institutions reconfigure legal consciousness over time, moving actors between claiming, strategic withdrawal, and renewed claiming.

## 5 Conclusion: toward a relational, dynamic, and mediated framework

This article has proposed a multi-layered conceptual framework to revitalize legal consciousness research by making its theoretical and socio-legal added value explicit. Rather than extending classic narrative approaches through another typology, it reconceptualizes rights consciousness as a mechanism-guided and cross-layer phenomenon. Digitalization, geopolitical positioning, and biographical trajectories do not operate as background context; they are constitutive conditions that reshape cognition, affect, and action.

In doing so, the framework addresses a central limitation of existing accounts: they often under-specify how similar foundational narratives yield divergent strategies of claiming, withdrawal, critique, and endurance under conditions of mediated information exposure, mass mobilization, and institutional disappointment.

The contribution to sociology of law is fourfold. First, the article shifts the unit of analysis from static narrative positions to relational and mechanism-centered processes. Second, it clarifies the distinction between legal consciousness and rights consciousness by locating rights-claiming within, rather than outside, broader legality-making. Third, it links legal consciousness more directly to rights mobilization, legal pluralism, recursive institutional change, and digital platform governance. Fourth, it identifies digital infrastructures, geopolitical benchmarking, and biographical temporality as constitutive conditions that pattern whether legality is experienced as claimable, insufficient, estranging, or strategically withdrawable.

- Relational: Consciousness does not reside solely within the individual but is co-constructed in dynamic interactions with others—be they perpetrators, state agents, social movements, or imagined communities (Liu, 2026; Chua and Engel, 2019).
- Temporally Evolving: It is not a static position but a biographical process, profoundly shaped by formative encounters with legal and political power that reconfigure an individual's relationship to their rights and identity over time (Tenorio, 2025).
- Affectively Mediated: It is deeply influenced by media, especially digital platforms, which shape not only knowledge but also the affective tone, political trust, and emotional landscape in which law is understood and experienced (Bailey et al., 2025; Peng et al., 2025).
- Globally Reflexive and Culturally Intimate: In an interconnected world, it can involve an awareness of one's group status vis-à-vis global norms and the use of cultural intimacy to manage discrepancies between legal ideals and lived realities; this mechanism is analytically strongest in semi-peripheral and post-transitional settings, where external benchmarking is pervasive (Fossum, 2025; Herzfeld, 2005).
- Paradoxical: Dynamics that undermine institutional trust can still intensify legality-oriented demands; resistance may reaffirm law's normative centrality; and increased algorithmic exposure can generate cognitive distortion alongside trust-mediated pathways (Feddersen et al., 2024; Peng et al., 2025).

The implications for future research are therefore not only thematic but methodological. Proposition 1 can be tested through survey, panel, or experimental designs that measure information exposure, platform use, exposure to official channels, moderation experiences, perceived opacity/fairness, political trust, and claim orientation, while explicitly addressing endogeneity and selection into platforms and channels. Proposition 2 invites longitudinal and protest-event research on how mobilization and organizational support reshape efficacy and rights talk over time. Proposition 3 can be tested by distinguishing legal insufficiency from alienation, estrangement, cynicism, and procedural injustice in interview, survey, or comparative case-study designs; one practical strategy is to pair items on perceived under-enforcement or selective impunity with items capturing continued normative commitment to legality. Proposition 4 calls for cross-national and within-country comparison across core, semi-peripheral, and post-transitional settings. Proposition 5 is best addressed through biographical and longitudinal designs tracking repeated encounters with welfare, policing, migration, or court institutions. Across political contexts, however, these indicators should not be assumed to be measurement-equivalent: under authoritarian information controls, heavy censorship, or platform capture, researchers may need to adapt what counts as official channels, moderation experiences, visible claims, or institutional trust. The framework's contribution is primarily analytic rather than prescriptive. Even so, it has bounded practical implications: people-centered justice design should attend to how digital interfaces distribute procedural burdens and trust, while platform governance should treat moderation, ranking, and opacity as socio-legal infrastructures that shape whether grievances become visible, intelligible rights claims or strategic withdrawal (Katsh and Rabinovich-Einy, 2017; Gillespie, 2018; Pasquale, 2015).
